# The Biomarker and Therapeutic Potential of Circular Rnas in Schizophrenia

**DOI:** 10.3390/cells9102238

**Published:** 2020-10-04

**Authors:** Artem Nedoluzhko, Natalia Gruzdeva, Fedor Sharko, Sergey Rastorguev, Natalia Zakharova, Georgy Kostyuk, Vadim Ushakov

**Affiliations:** 1 Faculty of Biosciences and Aquaculture, Nord University, PB 1490. 8049 Bodø, Norway; 2 Mental-Health Clinic No. 1 Named after N.A. Alexeev, Moscow Healthcare Department, Zagorodnoye Highway, 2, 115191 Moscow, Russia; nataliza80@gmail.com (N.Z.); kgr@yandex.ru (G.K.); tiuq@yandex.ru (V.U.); 3 National Research Center “Kurchatov Institute”, 1st Akademika Kurchatova Square, 123182 Moscow, Russia; nmg76@mail.ru (N.G.); fedosic@gmail.com (F.S.); rastorgueff@gmail.com (S.R.); 4 Research Center of Biotechnology of the Russian Academy of Sciences, Leninsky prospect 33/2, 119071 Moscow, Russia; 5 Institute for Advanced Brain Studies, Lomonosov Moscow State University, Leninskiye Gory, 119899 Moscow, Russia

**Keywords:** circular RNAs, circRNAs, microRNAs, RBPs, sponge, expression, biomarkers, therapy, schizophrenia

## Abstract

Circular RNAs (circRNAs) are endogenous, single-stranded, most frequently non-coding RNA (ncRNA) molecules that play a significant role in gene expression regulation. Circular RNAs can affect microRNA functionality, interact with RNA-binding proteins (RBPs), translate proteins by themselves, and directly or indirectly modulate gene expression during different cellular processes. The affected expression of circRNAs, as well as their targets, can trigger a cascade of events in the genetic regulatory network causing pathological conditions. Recent studies have shown that altered circular RNA expression patterns could be used as biomarkers in psychiatric diseases, including schizophrenia (SZ); moreover, circular RNAs together with other cell molecules could provide new insight into mechanisms of this disorder. In this review, we focus on the role of circular RNAs in the pathogenesis of SZ and analyze their biomarker and therapeutic potential in this disorder.

## 1. Introduction

The rapid development of novel DNA and RNA sequencing technologies has shed light on the mechanisms of multifactorial disorders, including their probability, origin, development, and pathogenesis [1,2,3].

Schizophrenia (SZ) is a serious psychiatric disorder that is caused by both environmental and molecular factors [4]. This disease seems to have accompanied humankind throughout history [5,6], despite findings that SZ risk alleles are under negative selection pressure and have been gradually removed from modern human populations [7]. Most often, this disease affects molecular pathways in cells that are related to brain development and, as a result, disrupts their functioning. The clinical picture of SZ is heterogeneous, and there are several classifications of this disorder based on the range and severity of symptoms [8,9].

In 2020, the number of published studies aimed at the molecular portrait of SZ has exceeded tens of thousands. However, a significant number of genes, molecular mechanisms, and pathways that are involved in SZ origin and pathogenesis still remain unknown or unclear [8]. Understanding the contribution of hidden molecular precursors and molecular mechanisms of SZ would undoubtedly help to provide better diagnostic and treatment options for this psychiatric disorder [10].

Genome-wide association studies (GWASs) have described only approximately 200 genome SNPs’ susceptibility loci for SZ [11]. Recent studies have shown that gene expression dysregulation, which is triggered by a number of epigenetic factors and aberrant expression of non-coding RNAs, could be one of the key causes of SZ and other psychiatric disorders [12]. 

It has long been considered that non-coding RNAs (ncRNAs), initially found in eukaryotes, are nothing more than non-functional “junk” RNAs accumulated during the long evolutionary process. Nowadays, many studies have shown that a significant part of this genomic “dark matter” could affect RNA silencing and post-transcriptional regulation of gene expression. As a result of recent advances in high throughput sequencing technologies, thousands of transcribed non-coding RNAs have been described in the vast majority of eukaryotic organisms [13]. These molecules play an important role in brain function [14,15] and are considered to be molecular markers or even to be a cause of psychiatric and mental disorders, such as SZ [16,17].

**Figure 1:**
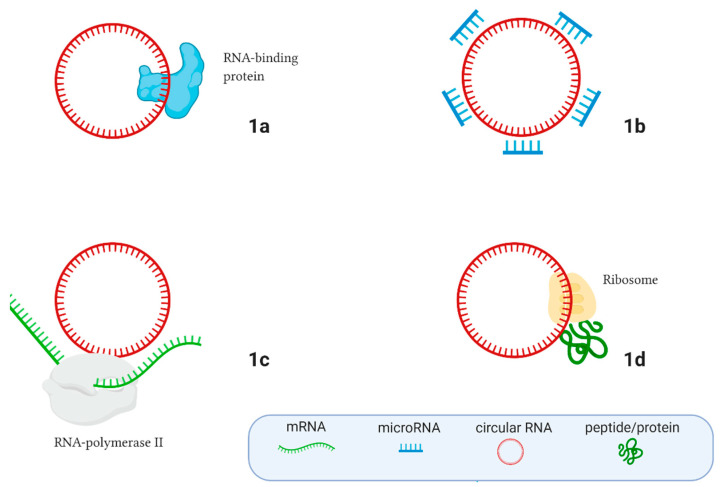
The main regulatory functions of circular RNAs in the cell. (**A**) Interacting with RNA-binding proteins; (**B**) Moderating microRNA expression, acting like microRNA sponges; (**C**) Interacting with RNA polymerase II and regulating gene expression; (**D**) Peptide/protein translation. Created with BioRender.com.

Circular RNAs (circRNAs) are most frequently non-coding, endogenous, single-stranded RNA molecules that play an important role in cell life. CircRNAs are covalently closed RNA molecules and often originate from protein-coding genes. Previously, it was thought that circular RNAs result from non-functional splicing errors that sometimes occur during the post-transcriptional processes in cells [18]. Currently, exonic, intronic, and exon-intronic circRNAs have been described in eukaryotes, but only a few of their functions have been revealed [19,20], and circRNAs remain almost entirely unexplored RNA molecules. The main circRNA cell functions are shown in Figure 1. CircRNAs can work like sponges that contain targets for RNA binding proteins (Figure 1a) and microRNAs (Figure 1b), thereby participating in gene expression regulation. They can also regulate gene expression by interacting with RNA polymerase II (Figure 1c), and can even produce peptides and proteins (Figure 1d) [19,20].

Recent studies have shown a significant impact of circRNAs on the origin and development of SZ, as well as an important role of circRNAs in other non-coding and protein-coding gene expression regulation [21]. Recently, Li and colleagues briefly reviewed circRNAs potential as diagnostic biomarkers for SZ and depression [22]. Here, we provide a more detailed review of the study results focused on the role of microRNAs (miRNAs), mRNAs, RNA binding proteins (RBPs) activity, and circRNA in SZ and discuss the potential implications of circRNAs as an important part of cell machinery, including new insights into the therapeutic potential of artificially engineered circular RNAs.

## 2. The Role of Circular Rnas Differential Expression in Schizophrenia

Molecular genetic data for circRNAs’ activity in SZ remain limited, despite significant progress made in functional and prediction analysis for this type of RNA [23,24,25]. To date, there have only been a few studies published that have described the potential role of circRNAs in psychiatric disorders, such as SZ.

One analysis of dorsolateral prefrontal cortex (DLPFC) of postmortem healthy and schizophrenic human brains showed significant changes in circRNA expression. A comparison of schizophrenic individuals and healthy controls resulted in the identification of 574 differentially expressed circRNAs, and more than two-thirds of them (390, 68%) were downregulated in SZ. Most importantly, the vast majority of these differentially expressed circular RNAs had miRNA targets and were considered to be potential sponges for microRNAs regulating their activity in the brain [26]. However, differential expression between SZ cases and healthy controls was not observed in another study that studied the circRNA expression in DLPFC [27].

It has been also shown that neuronal-enriched circHomer1a (host gene, Homer protein homolog 1) apparently played an important role in cognition processes, as its expression was downregulated in SZ postmortem prefrontal cortex (PFC) area of the human brain, while an intronic circCUL4A (host gene, ubiquitin ligase Cullin-4A) was significantly upregulated [28].

According to the available data, circRNAs can be used as biomarkers in SZ. Comparative analysis of circRNA expression in patients living with SZ has shown that after one- and two-month treatments and symptom improvements, the circular RNA hsa_circRNA_104597 (host gene, *PLEKHA2*) was significantly upregulated in peripheral blood mononuclear cells transcriptome [29].

A comparative analysis of circRNAs that were extracted from blood plasma exosomes also showed significant differences between healthy and schizophrenic patients. Tan et al. identified 44 differentially expressed circRNAs using deep sequencing. Eight of the circRNAs were validated using real-time PCR, and the targets for microRNAs were then bioinformatically predicted for four of them [30]. Interestingly, one of the detected highly expressed circRNA, i.e., has_circ, chr5_69175537_69174877_+660, carried targeted sequences for miR-34a-5p and miR-449a molecules, which had been previously validated as a good prognostic model for SZ [31].

## 3. The Role of Differential Gene Expression in SchizophreniA: Circular Rnas as Participants and Regulators of Gene Expression

A significant number of case–control SZ studies have presented controversial results with a small number of identified genes that had either up- or downregulated expression, possibly related to SZ [32]. These results were often inconsistent with molecular pathway studies because of several issues related to gene ontology analysis [33]; therefore, a number of genes and their protein products remained without an identified function [34].

It has been shown that SZ has mostly been related to the loss of molecular pathways functionality and change of the protein-coding gene expression through the dysregulation of non-coding RNA expression, methylation, novel SNPs, structural variants (SNVs), and even environment influence during fetal development and in the lifetime of the individuals [35]. In the last decade, hundreds of studies have been published that described a number of protein-coding genes that were possibly involved in the origin and development of SZ [36]. The alterations of protein-coding gene expression also have an important impact on this mental disorder, primarily in the prefrontal cortex. Several biological pathways associated with synaptic function and signaling pathways have been shown to be disrupted in SZ [37,38].

Moreover, the comprehensive comparative analysis of gene expression in the hippocampus, DLPFC, and dorsal striatum (DS) of healthy and schizophrenic human brains showed significant differences in gene expression [39]. At least 40 genes have been described as differentially expressed during the joint analysis of three brain regions known as affected in SZ [40]. Most of these genes (32 out of 40 genes) were downregulated in schizophrenic human brains; moreover, these alterations in gene expression have the same nature of expression changes. The classification of these genes based on their molecular functions showed that they were involved in common molecular pathways related to neuronal cell life cycle (e.g., nucleic acid binding transcription factor activity, receptor-ligand binding, structural molecule activity, etc.) [39].

It should be noted that differential expression of protein-coding genes in SZ has a polygenic nature, and there are differences in gene expression even in whole blood between healthy controls, patients with high clinical risk for psychosis, first episode of psychosis, and chronic schizophrenic patients. Particularly, myelin basic protein (*MBP*) and NudE neurodevelopment protein 1 like 1 (*NDEL1*) genes were upregulated in the first episode of psychosis, as compared with other groups, and were possibly related to the first molecular signs of SZ. At the same time, the DiGeorge syndrome critical region gene (*DGCR2*) was downregulated in schizophrenic individuals as compared with healthy controls and the first episode of psychosis. Possibly, low expression of *DGCR2* in SZ could reflect antipsychotic treatment or a disorder stage [41].

Recent studies have shown the new fact that circRNAs contained short open reading frames and as a result could produce peptides that play an important role in cell processes (e.g., circ-ZNF609 in myogenesis [42]). At the same time, circ-ZNF609 itself could become a critical factor in major human diseases, such as cancer [43,44,45]. Another study showed that artificial circular RNAs could be expressed in the cell in the same manner as a protein-coding linear RNA. However, poly(A) and poly(U) highly repeat regions could be reduced in circular mRNA translation. The high level of circRNA stability should be promoting a new tool for long-time gene expression in modified cells and tissues [46].

Currently, there has not been enough data about circRNAs that produced peptides that could take part in the mechanisms of the origin and development of SZ, but recently shown involvement of these molecules in the pathogenesis of cancer has suggested this possibility. We assume that subsequent research in this direction would shed light on circRNA translation in the brains of patients with SZ, inter alia, using mechanisms similar to rolling circle amplification [47,48].

However, circular RNAs produce peptides and proteins, and participate in the transcription regulation of other genes through the mechanism, namely, “mRNA traps” [49]. Moreover, this participation can occur both at the transcription initiation stage and at the RNA elongation stage [50].

Currently, only one reliable study that suggests the participation of circRNAs in the initiation of translation of other genes has been published [51]. It was shown that some exon-intronic circRNAs could enhance gene expression by affecting parental genes at the initiation stage. Possibly, exon-intron circular RNAs (circEIF3J and circPAIP2) can create a functional complex with U1 small nuclear ribonucleoproteins and interact with RNA polymerase II at the gene promoter and assist gene expression. It has been shown that the knockdown of these two circular RNAs decreased expression of their parental genes, eukaryotic translation initiation factor 3 subunit J (*EIF3J*), and polyadenylate-binding protein-interacting protein 2 (*PAIP2*). In addition, 111 other covalently closed RNA molecules were bioinformatically predicted to be potential translation initiators, but experimentally (out of 15 exon-intronic circRNAs), this possibility was proven for only two of them, circEIF3J and circPAIP2 [51]. This study remains among the few studies that have described the role of circRNAs in the initiation of transcription [51], and have worked on the influence of circular RNAs on the process of elongation during transcription [52].

Thus, despite the extensive functional connection between circRNAs and protein-coding genes, the results published to date have not been adequately analyzed in SZ studies. Moreover, in some studies, where rRNA depletion was used for transcriptomic analysis, circular RNA reads were included as a part of the differential expression analysis of protein-coding genes [53].

## 4. The Role of Mirnas in SchizophreniA: Circular Rnas as Sponges for Micrornas

miRNAs are short (~22 nucleotides), single-stranded, endogenous ncRNA molecules with different regulatory functions. miRNAs play a significant role in modulating gene expression from translation inhibition to mRNA degradation during different cell processes (e.g., cell differentiation, apoptosis, and proliferation) in plants and animals [54,55,56,57]. Recently, miRNA-mediated gene expression has gained research interest in relation to the pathology of psychiatric diseases. Interacting with their target genes in different brain areas, miRNAs play diverse roles in brain evolution and development [58,59,60]. Moreover, these molecules are crucial for brain functions, especially during the fetal period [61], when maternal stress can induce psychiatric disorders in offspring [62].

It has been previously shown that miRNAs have an important effect on SZ susceptibility, but the study results have varied depending on sampling size, brain area, ethnicity, and other factors. Particularly, the expression level of miR-106b and its significance and data reproducibility have been discussed in several publications [63,64,65].

Analysis of miR-346 expression, which is encoded within intron 2 of the glutamate receptor ionotropic delta 1 (*GRID1*) gene implicated in SZ susceptibility, showed that this short RNA is downregulated in SZ in the DLPFC [66]. Moreover, this miRNA can be used as a blood diagnostic marker in the context of this disorder [67]. Other promising results have been obtained in the overexpression experiments with miR-92b, miR-495, and miR-134 in the DLPFC of postmortem brains. Their involvement in pathophysiology networks was demonstrated through the repression of *BCL11A*, *PLP1*, and *SYT11* genes responsible for neurodevelopment, particularly synaptic development [68], and oligodendrocyte functions [69]. Interestingly, miR-92 was shown to be downregulated in the PFC, similar to 14 other miRNAs in the schizophrenic human brain, while miR-106b expression was upregulated [65].

The SNPs in miRNA genes, as well as in their target sites, can also have a significant impact on SZ development [70]. SNPs in miR-137 are the classical example of miRNA mutations directly associated with an increased risk of SZ [71,72].

Some miRNAs can be used as biomarkers for SZ even when they have an unaltered expression level in the brain, for example, miR-130b has an increased expression level in the blood, not in the brain [73]. To date, numerous articles that aimed at looking for circulating miRNA markers in the blood have been published [31,67,74]. Dozens of miRNAs are considered to be potential markers of SZ, but the list of these regulatory molecules varies depending on the different factors such as the stage of the disorder [75].

Many reports have shown that microRNAs could be used as useful biomarkers, for example, miR-34a, miR-449a, miR-564, miR-432, miR-548d, miR-572, and miR-652 have been described as possible biomarkers with altered expression in the peripheral blood of schizophrenic patients [74]. Moreover, these expression patterns did not change even after hospitalization with treatment [76]. Aberrant expression of miR-34a-5p, miR-432-5p, and miR-449a molecules has also been validated as a good prognostic model for SZ in subsequent studies [31]. Apart from the above-mentioned microRNAs, miR-181b-5p, miR-21-5p, miR-195-5p, and miR-346 could also be considered as biomarkers with high diagnostic sensitivity in peripheral blood mononuclear cells [67].

Despite a significant number of studies related to aberrant miRNA expression in the brain, its association with the origin and development of SZ, and the possibility of using altered miRNA expression in the peripheral blood cells as a biomarker of SZ, there are currently no clear biomarkers for any SZ type [74].

Recent studies have shown that there are several mechanisms that direct silenced microRNA expression. One mechanism was related to antagomirs, chemically engineered oligonucleotides [77], which showed effectivity as a novel therapeutic tool in temporal lobe epilepsy through the silencing of miR-134 in the mouse model [78].

Another mechanism involves circRNAs and can be found mostly in the eukaryotic cells. miRNA activity has been shown to be affected by circRNAs, which contain miRNAs targets and work like a sponge by modulating their activity [21]. Moreover, circRNAs can both enhance the expression of a targeted gene by aggregating its antagonist microRNAs or inhibit target gene expression by releasing previously aggregated microRNAs again when the circRNA molecule is cleaved [79]. Circular RNAs have a significant number of target sites for the specific microRNAs and can effectively interact with them [21].

It has been previously shown that circRNAs regulate transcription by actively binding miRNAs [51]. Examples include Cdr1as, which binds miR-7 and miR-671 in human and mouse brains [80], hsa_circ_0088036, which regulates rheumatoid arthritis progression via sponging miR-140-3p and upregulating silent information regulator 1 (*SIRT 1*) expression [81], and circ-YOD1, which negatively regulates expression of miR-21-3p and miR-296-3p in coronary artery disease patients [82]. 

Similar to RBP–circRNA interactions, significant progress has been achieved in studies about microRNA sponging by circular RNAs in different types of cancer. These studies mainly described specific human circular RNA and its potential microRNA target. The vast majority of these investigations has been published during the last two years and describes the role of miRNA sponging in breast [83], lung [84], colon [85], and other cancer types [86,87]. Interestingly, some of them, for example, hsa_circ_0009910, potentially play a role in a wide range of malignancies [88].

These and other results demonstrate that microRNA sponging is an effective molecular method for miRNA expression regulation in the cell. However, to date, no data about circRNA’s role as a sponge for microRNAs in SZ pathogenesis have been published, although their involvement in the origin and development of SZ is suggested [30].

## 5. The Role of Rna Binding Proteins (Rbps) in SchizophreniA: Circular Rnas as Sponges for Rbps

RNA binding proteins (RBPs) are a diverse group of proteins located in the nucleus and cytoplasm. For a long time, it was supposed that these molecules worked as post-transcriptional regulators of RNA expression, but more recent studies have shown the flip side of these RNA–protein interactions, in which RNA transcripts regulated protein function by forming ribonucleoprotein complexes [89].

The multiple possibilities of gene expression regulation through RBPs create a significant chapter of the modern molecular biology and describe the modifications of cell molecular pathways, including RBP involvement in serious disorders, such as cancer [90] and neurodegenerative [91] and psychiatric [92] diseases. Despite significant progress in studying RBP involvement in neuronal development [93,94,95] and RBPs’ influence on the expression of thousands of brain mRNAs [96], there are only a few studies that have found a functional role of RBPs in SZ [97].

One of the first cases with RBPs and SZ was described with the *disrupted in Schizophrenia 1 (DISC1*) gene, which is known as a target for hematopoietic zinc finger (HZF) and several other RNA-binding proteins [98]. *DISC1* plays a significant role in neurogenesis, and synaptic plasticity and translocations within *DISC1* are related to SZ [99].

The zinc finger protein 804A (*ZNF804A*) gene is broadly expressed in brain tissues and has been suggested to be one of the possible SZ risk factors [100]. *ZNF804A* has DNA, RNA, and protein binding ability and acts as a regulator of gene expression [101,102]. Several GWASs have shown that mutations in this gene had a strong association with SZ [103,104].

TDP-43 and NOVA-1 RNA-binding proteins have also been described as regulators of the alternative splicing of the *TRAF2* gene and the NCK interacting kinase (*TNIK*) gene, which is known as an important factor for neuronal cell differentiation and as a risk factor in SZ [105].

Thus, the examples presented above show a deep relationship between the origin, development, and pathogenesis of SZ and RBP activity in brain tissues. Moreover, it seems that circRNAs can also have an important impact in these processes as a part of molecular pathways, since they can bind to RBPs, thereby regulating the effectiveness of their work [92].

Circular RNAs are able to interact with a significant number of RBPs in the cytoplasm; those proteins are involved in the biogenesis of circRNAs and work hand in hand in various cellular pathways, similar to other protein-coding and non-coding RNAs [106]. Currently, the role of such interactions is poorly understood and the significance of such interactions is still under discussion, given the relatively low concentration of circRNAs in the cells [79]. Existing bioinformatical tools allow us to identify circRNA–protein interactions using neural networks [107] and even in previously generated cross-linking immunoprecipitation sequencing (CLIP-Seq) datasets [108].

It is expected that RBP–circRNA interactions play an important role in the origin and pathogenesis of different polygenic disorders, including different types of cancer. Particularly, it has been shown that SCD-circRNA 2, which was derived from the stearoyl-CoA desaturase, was highly expressed in hepatocellular carcinoma tissue and correlated with poor patient prognosis. Moreover, this type of ncRNA expression was dynamically regulated by RNA-binding protein 3 (RBM3) that had an important impact on SCD-circRNA 2 expression in tumor cells [109]. In addition, a recent study showed that circ0005276 circular RNA possibly interacted with FUS binding protein (*FUS*); this RBP-circRNA complex could dysregulate X-linked inhibitor of apoptosis protein (*XIAP*) transcription, and thereby influence the development of prostate cancer [110].

Additionally, circfoxo3-p21-CDK2 complex that contains circFoxo3 circRNA, cyclin-dependent kinase inhibitor 21 (*p21*), and cyclin-dependent kinase 2 (*CDK2*) inhibited mouse cancer progression [111].

Recently, using bioinformatical prediction, it has been shown that neuronal HuD protein (ELAVL4) contained AU-rich elements that were attractive for hundreds of mice brain-expressed circRNAs. RNA immunoprecipitation confirmed this finding and showed that approximately 600 mice brain-expressed circRNAs could interact with HuD [112]; moreover, 226 of them originated from the previously described circHomer1a gene [28].

Unfortunately, despite the high level of SZ morbidity in different countries (approximately 1.45%), the mechanisms of RNA binding proteins and circular RNA complex interactions and their functional role in SZ pathogenesis remain unclear. A small number of studies that evaluated the differential expression of this type of ncRNAs in the human brain [26,27,28,29] have demonstrated only the huge potential of these studies.

## 6. Concluding Remarks: Perspectives of Circular Rnas Therapy

The previous review sections have clearly demonstrated that, despite a number of molecular differences between healthy and schizophrenic humans, there was no single causal marker for this psychiatric disorder. Apparently, the origin and development of SZ are caused by a disruption of several molecular pathways, or even networks, at once [113]. 

It is most likely that the cascading disruption of molecular pathways during SZ development is similar to the domino effect, where the dysregulation of one molecular process sets off the subsequent ones. Most of the studies available to date comparatively describe one or two types of molecular markers in search for molecular mechanisms of SZ. Usually, the differences are found, but most results only raise additional questions [114].

Circular RNAs are becoming a relevant research topic and, for several years, have attracted significant interest in molecular studies [18]. It is now well known that they are involved in the regulation of the expression of other non-coding RNAs and protein-coding genes, and are able to interact with the protein machinery of the cell and independently translate proteins [18]. The significant amount of molecular data accumulated to date, the development of deep sequencing platforms as well as the robotization of many technological processes, and the development of machine learning methods have opened up new opportunities for understanding the genetic and epigenetic causes of the aspects and development of polygenic disorders [115].

**Figure 2:**
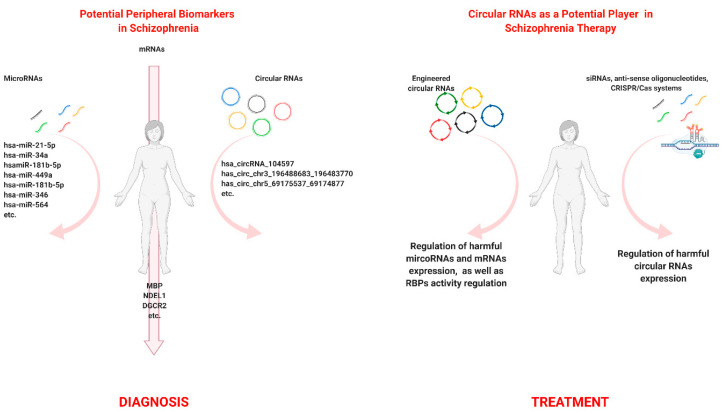
The biomarker and therapeutic potential of circular RNAs in schizophrenia. (**A**) Potential molecular markers in schizophrenia. (**B**) Circular RNAs as a potential player in schizophrenia therapy. Created with BioRender.com.

We suppose that such diversity in circRNA functionality makes this type of RNA, together with other molecules, a promising biomolecular marker (Figure 2a), which would help to explain molecular processes underlying the SZ. CircRNAs and microRNA have become crucial molecular markers in SZ studies. The important role of miRNAs in the cell molecular pathways has been known for a relatively long time. These regulatory short ncRNAs are believed to manage expression of 20 to 60 percent of protein-coding genes in mammalian genomes [116,117,118]. At the same time, the dysregulation of miRNA expression can cause a number of psychiatric disorders, including SZ [12]. For a long time, miRNAs have been considered to be potential therapeutic agents themselves [119]. However, the low targeting specificity [117] and high degradation rate in the cells and tissues [120] turn their potential implementation in SZ therapy into a complicated issue. In addition, circular RNAs have a significant advantage due to their resistance to exonuclease-mediated degradation (two to five times more stable than linear RNAs) and have a tendency to accumulate in cells with slow division rates, such as neurons, where they are often localized in dendrites, near synapses [121,122,123], which makes them more suitable for different types of therapies directed at the regulation of miRNA or mRNA expression.

It is important to note that, despite several pros, circRNAs have some cons related to their slow biogenesis in vivo, which, however, can be accelerated via RNA polymerase II activity [122]. Circular RNAs that have poly(adenosine) or poly(thymidine) in 3′ UTR are also expressed non-effectively, unlike their linear forms [46].

It has been suggested that circRNAs have diagnostic value and could be used as potential therapeutic agents against polygenic diseases (Figure 2). Currently, it seems that up-to-date knowledge about these molecules has resulted in the development of artificially engineered circular RNA [124]. Particularly, Lavenniah et al. developed circRNA sponges that inhibited the activity of cardiac pro-hypertrophic microRNAs (miR-132 and miR-212). These artificial circRNAs were engineered and successfully used in vivo on a cardiac hypertrophy mouse model [125].

circRNAs themselves can be considered to be potential targets in schizophrenia. Therefore, studies analyzing circRNAs that disrupt normal molecular pathways and cause SZ also play an important role. Such potentially harmful, upregulated circRNAs can be silenced by short interfering RNAs (siRNAs), by anti-sense oligonucleotides, and finally by using genome editing methods, for example, the CRISPR/Cas system (Figure 2b) [126].

The importance of research and development advances in the molecular therapy for SZ cannot be overestimated, since SZ is the most common variant of psychiatric disorders with cognitive and sensory symptoms and a prominent hereditary component. We suppose that future studies should focus on a deep search for prognostic biomarkers based on genomic, transcriptomic, and epigenetic data and focus on the development of molecular tools and delivery systems to target tissues and organs.

## References

[1] Gurdasani D., Barroso I., Zeggini E., Sandhu M.S. (2019). Genomics of disease risk in globally diverse populations. Nat. Rev. Genet..

[2] Esplin E.D., Oei L., Snyder M.P. (2014). Personalized sequencing and the future of medicine: Discovery, diagnosis and defeat of disease. Pharmacogenomics.

[3] Lightbody G., Haberland V., Browne F., Taggart L., Zheng H., Parkes E., Blayney J.K. (2019). Review of applications of high-throughput sequencing in personalized medicine: Barriers and facilitators of future progress in research and clinical application. Brief. Bioinform..

[4] Ershova E.S., Jestkova E.M., Martynov A.V., Shmarina G.V., Umriukhin P.E., Bravve L.V., Zakharova N.V., Kostyuk G.P., Saveliev D.V., Orlova M.D. (2019). Accumulation of Circulating Cell-Free CpG-Enriched Ribosomal DNA Fragments on the Background of High Endonuclease Activity of Blood Plasma in Schizophrenic Patients. Int. J. Genomics.

[5] Prufer K., de Filippo C., Grote S., Mafessoni F., Korlevic P., Hajdinjak M., Vernot B., Skov L., Hsieh P., Peyregne S. (2017). A high-coverage Neandertal genome from Vindija Cave in Croatia. Science.

[6] Srinivasan S., Bettella F., Mattingsdal M., Wang Y., Witoelar A., Schork A.J., Thompson W.K., Zuber V., Winsvold B.S., Zwart J.A. (2016). Genetic Markers of Human Evolution Are Enriched in Schizophrenia. Biol. Psychiatry.

[7] Liu C., Everall I., Pantelis C., Bousman C. (2019). Interrogating the Evolutionary Paradox of Schizophrenia: A Novel Framework and Evidence Supporting Recent Negative Selection of Schizophrenia Risk Alleles. Front. Genet..

[8] Owen M.J., Sawa A., Mortensen P.B. (2016). Schizophrenia. Lancet.

[9] Picchioni M.M., Murray R.M. (2007). Schizophrenia. Bmj-Brit. Med. J..

[10] Gejman P.V., Sanders A.R., Kendler K.S. (2011). Genetics of Schizophrenia: New Findings and Challenges. Annu. Rev. Genom. Hum. Genet..

[11] Huo Y., Li S., Liu J., Li X., Luo X.J. (2019). Functional genomics reveal gene regulatory mechanisms underlying schizophrenia risk. Nat. Commun..

[12] Gibbons A., Udawela M., Dean B. (2018). Non-Coding RNA as Novel Players in the Pathophysiology of Schizophrenia. Noncoding RNA.

[13] Palazzo A.F., Lee E.S. (2015). Non-coding RNA: What is functional and what is junk?. Front. Genet..

[14] He Z., Bammann H., Han D., Xie G., Khaitovich P. (2014). Conserved expression of lincRNA during human and macaque prefrontal cortex development and maturation. RNA.

[15] Zhang B., Han D., Korostelev Y., Yan Z., Shao N., Khrameeva E., Velichkovsky B.M., Chen Y.P., Gelfand M.S., Khaitovich P. (2016). Changes in snoRNA and snRNA Abundance in the Human, Chimpanzee, Macaque, and Mouse Brain. Genome Biol. Evol..

[16] Beveridge N.J., Gardiner E., Carroll A.P., Tooney P.A., Cairns M.J. (2010). Schizophrenia is associated with an increase in cortical microRNA biogenesis. Mol. Psychiatr..

[17] Safari M.R., Komaki A., Arsang-Jang S., Taheri M., Ghafouri-Fard S. (2019). Expression Pattern of Long Non-coding RNAs in Schizophrenic Patients. Cell Mol. Neurobiol..

[18] Holdt L.M., Kohlmaier A., Teupser D. (2018). Molecular roles and function of circular RNAs in eukaryotic cells. Cell Mol. Life Sci..

[19] Memczak S., Jens M., Elefsinioti A., Torti F., Krueger J., Rybak A., Maier L., Mackowiak S.D., Gregersen L.H., Munschauer M. (2013). Circular RNAs are a large class of animal RNAs with regulatory potency. Nature.

[20] Yu C.Y., Kuo H.C. (2019). The emerging roles and functions of circular RNAs and their generation. J. Biomed. Sci..

[21] Hansen T.B., Jensen T.I., Clausen B.H., Bramsen J.B., Finsen B., Damgaard C.K., Kjems J. (2013). Natural RNA circles function as efficient microRNA sponges. Nature.

[22] Li Z., Liu S., Li X., Zhao W., Li J., Xu Y. (2020). Circular RNA in Schizophrenia and Depression. Front. Psychiatry.

[23] Shanmugapriya, Huda H.A., Vijayarathna S., Oon C.E., Chen Y., Kanwar J.R., Ng M.L., Sasidharan S. (2018). Functional Analysis of Circular RNAs. Adv. Exp. Med. Biol..

[24] Hansen T.B., Veno M.T., Damgaard C.K., Kjems J. (2016). Comparison of circular RNA prediction tools. Nucleic Acids Res..

[25] Nedoluzhko A., Sharko F., Rbbani M.G., Teslyuk A., Konstantinidis I., Fernandes J.M.O. (2020). CircParser: A novel streamlined pipeline for circular RNA structure and host gene prediction in non-model organisms. Peerj.

[26] Mahmoudi E., Fitzsimmons C., Geaghan M.P., Shannon Weickert C., Atkins J.R., Wang X., Cairns M.J. (2019). Circular RNA biogenesis is decreased in postmortem cortical gray matter in schizophrenia and may alter the bioavailability of associated miRNA. Neuropsychopharmacology.

[27] Liu Z., Ran Y., Tao C., Li S., Chen J., Yang E. (2019). Detection of circular RNA expression and related quantitative trait loci in the human dorsolateral prefrontal cortex. Genome Biol..

[28] Zimmerman A.J., Hafez A.K., Amoah S.K., Rodriguez B.A., Dell’Orco M., Lozano E., Hartley B.J., Alural B., Lalonde J., Chander P. (2020). A psychiatric disease-related circular RNA controls synaptic gene expression and cognition. Mol. Psychiatry.

[29] Yao G., Niu W., Zhu X., He M., Kong L., Chen S., Zhang L., Cheng Z. (2019). hsa_circRNA_104597: A novel potential diagnostic and therapeutic biomarker for schizophrenia. Biomark Med..

[30] Tan G., Wang L., Liu Y., Zhang H., Feng W., Liu Z. (2020). The alterations of circular RNA expression in plasma exosomes from patients with schizophrenia. J. Cell Physiol..

[31] He K., Guo C., He L., Shi Y. (2018). MiRNAs of peripheral blood as the biomarker of schizophrenia. Hereditas.

[32] Fromer M., Roussos P., Sieberts S.K., Johnson J.S., Kavanagh D.H., Perumal T.M., Ruderfer D.M., Oh E.C., Topol A., Shah H.R. (2016). Gene expression elucidates functional impact of polygenic risk for schizophrenia. Nat. Neurosci..

[33] Gaudet P., Dessimoz C. (2017). Gene Ontology: Pitfalls, Biases, and Remedies. Methods Mol. Biol..

[34] Ellens K.W., Christian N., Singh C., Satagopam V.P., May P., Linster C.L. (2017). Confronting the catalytic dark matter encoded by sequenced genomes. Nucleic Acids Res..

[35] Caspi A., Moffitt T.E. (2006). Gene-environment interactions in psychiatry: Joining forces with neuroscience. Nat. Rev. Neurosci..

[36] Kumarasinghe N., Tooney P.A., Schall U. (2012). Finding the needle in the haystack: A review of microarray gene expression research into schizophrenia. Aust. N. Z. J. Psychiatry.

[37] Barnes M.R., Huxley-Jones J., Maycox P.R., Lennon M., Thornber A., Kelly F., Bates S., Taylor A., Reid J., Jones N. (2011). Transcription and pathway analysis of the superior temporal cortex and anterior prefrontal cortex in schizophrenia. J. Neurosci. Res..

[38] Middleton F.A., Peng L., Lewis D.A., Levitt P., Mirnics K. (2005). Altered expression of 14-3-3 genes in the prefrontal cortex of subjects with schizophrenia. Neuropsychopharmacology.

[39] Kumar A., Pareek V., Singh H.N., Faiq M.A., Narayan R.K., Raza K., Kumar P. (2019). Altered Expression of a Unique Set of Genes Reveals Complex Etiology of Schizophrenia. Front. Psychiatry.

[40] Guo J.Y., Ragland J.D., Carter C.S. (2019). Memory and cognition in schizophrenia. Mol. Psychiatry.

[41] Ota V.K., Moretti P.N., Santoro M.L., Talarico F., Spindola L.M., Xavier G., Carvalho C.M., Marques D.F., Costa G.O., Pellegrino R. (2019). Gene expression over the course of schizophrenia: From clinical high-risk for psychosis to chronic stages. NPJ Schizophr..

[42] Legnini I., Di Timoteo G., Rossi F., Morlando M., Briganti F., Sthandier O., Fatica A., Santini T., Andronache A., Wade M. (2017). Circ-ZNF609 Is a Circular RNA that Can Be Translated and Functions in Myogenesis. Mol. Cell.

[43] Rossi F., Legnini I., Megiorni F., Colantoni A., Santini T., Morlando M., Di Timoteo G., Dattilo D., Dominici C., Bozzoni I. (2019). Circ-ZNF609 regulates G1-S progression in rhabdomyosarcoma. Oncogene.

[44] Zuo Y., Shen W., Wang C., Niu N., Pu J. (2020). Circular RNA Circ-ZNF609 Promotes Lung Adenocarcinoma Proliferation by Modulating miR-1224-3p/ETV1 Signaling. Cancer Manag. Res..

[45] Zhang M., Huang N., Yang X., Luo J., Yan S., Xiao F., Chen W., Gao X., Zhao K., Zhou H. (2018). A novel protein encoded by the circular form of the SHPRH gene suppresses glioma tumorigenesis. Oncogene.

[46] Wang Y., Wang Z. (2015). Efficient backsplicing produces translatable circular mRNAs. RNA.

[47] Abe N., Matsumoto K., Nishihara M., Nakano Y., Shibata A., Maruyama H., Shuto S., Matsuda A., Yoshida M., Ito Y. (2015). Rolling Circle Translation of Circular RNA in Living Human Cells. Sci. Rep..

[48] Chen C.Y., Sarnow P. (1995). Initiation of protein synthesis by the eukaryotic translational apparatus on circular RNAs. Science.

[49] Huang S., Yang B., Chen B.J., Bliim N., Ueberham U., Arendt T., Janitz M. (2017). The emerging role of circular RNAs in transcriptome regulation. Genomics.

[50] Bose R., Ain R. (2018). Regulation of Transcription by Circular RNAs. Adv. Exp. Med. Biol..

[51] Li Z., Huang C., Bao C., Chen L., Lin M., Wang X., Zhong G., Yu B., Hu W., Dai L. (2015). Exon-intron circular RNAs regulate transcription in the nucleus. Nat. Struct. Mol. Biol..

[52] Zhang Y., Zhang X.O., Chen T., Xiang J.F., Yin Q.F., Xing Y.H., Zhu S., Yang L., Chen L.L. (2013). Circular intronic long noncoding RNAs. Mol. Cell.

[53] Zhao S., Zhang Y., Gamini R., Zhang B., von Schack D. (2018). Evaluation of two main RNA-seq approaches for gene quantification in clinical RNA sequencing: polyA+ selection versus rRNA depletion. Sci. Rep..

[54] Bartel D.P. (2004). MicroRNAs: Genomics, biogenesis, mechanism, and function. Cell.

[55] Rastorguev S.M., Nedoluzhko A.V., Gruzdeva N.M., Boulygina E.S., Sharko F.S., Ibragimova A.S., Tsygankova S.V., Artemov A.V., Skryabin K.G., Prokhortchouk E.B. (2017). Differential miRNA expression in the three-spined stickleback, response to environmental changes. Sci. Rep..

[56] Shulga O.A., Nedoluzhko A.V., Shchennikova A.V., Gruzdeva N.M., Shelenkov A.A., Sharko F.S., Sokolov A.S., Pantiukh E.S., Rastorguev S.M., Prokhortchouk E.B. (2017). Profiling of microRNAs in wild type and early flowering transgenic Chrysanthemum morifolium by deep sequencing. Plant. Cell Tiss. Org..

[57] Zhang Q., Kopp M., Babiak I., Fernandes J.M.O. (2018). Low incubation temperature during early development negatively affects survival and related innate immune processes in zebrafish larvae exposed to lipopolysaccharide. Sci. Rep..

[58] Chen W., Qin C. (2015). General hallmarks of microRNAs in brain evolution and development. Rna Biol.

[59] Shao N.Y., Hu H.Y., Yan Z., Xu Y., Hu H., Menzel C., Li N., Chen W., Khaitovich P. (2010). Comprehensive survey of human brain microRNA by deep sequencing. BMC Genom..

[60] Somel M., Liu X., Khaitovich P. (2013). Human brain evolution: Transcripts, metabolites and their regulators. Nat. Rev. Neurosci..

[61] Tang Y., Liu D., Zhang L., Ingvarsson S., Chen H. (2011). Quantitative analysis of miRNA expression in seven human foetal and adult organs. PLoS ONE.

[62] Zucchi F.C., Yao Y., Ward I.D., Ilnytskyy Y., Olson D.M., Benzies K., Kovalchuk I., Kovalchuk O., Metz G.A. (2013). Maternal stress induces epigenetic signatures of psychiatric and neurological diseases in the offspring. PLoS ONE.

[63] Jeffries C.D., Perkins D.O. (2016). Reproducibility and Visual Inspection of Data. Biol. Psychiatry.

[64] Moreau M.P., Bruse S.E., David-Rus R., Buyske S., Brzustowicz L.M. (2011). Altered microRNA expression profiles in postmortem brain samples from individuals with schizophrenia and bipolar disorder. Biol. Psychiatry.

[65] Perkins D.O., Jeffries C.D., Jarskog L.F., Thomson J.M., Woods K., Newman M.A., Parker J.S., Jin J., Hammond S.M. (2007). microRNA expression in the prefrontal cortex of individuals with schizophrenia and schizoaffective disorder. Genome Biol..

[66] Zhu Y., Kalbfleisch T., Brennan M.D., Li Y. (2009). A MicroRNA gene is hosted in an intron of a schizophrenia-susceptibility gene. Schizophr. Res..

[67] Liu S., Zhang F., Wang X., Shugart Y.Y., Zhao Y., Li X., Liu Z., Sun N., Yang C., Zhang K. (2017). Diagnostic value of blood-derived microRNAs for schizophrenia: Results of a meta-analysis and validation. Sci. Rep..

[68] Schratt G.M., Tuebing F., Nigh E.A., Kane C.G., Sabatini M.E., Kiebler M., Greenberg M.E. (2006). A brain-specific microRNA regulates dendritic spine development. Nature.

[69] Santarelli D.M., Carroll A.P., Cairns H.M., Tooney P.A., Cairns M.J. (2020). Schizophrenia-associated MicroRNA-Gene Interactions in the Dorsolateral Prefrontal Cortex. Genom. Proteom. Bioinform..

[70] Sun X., Zhang J. (2014). Identification of putative pathogenic SNPs implied in schizophrenia-associated miRNAs. BMC Bioinform..

[71] Kuswanto C.N., Sum M.Y., Qiu A., Sitoh Y.Y., Liu J., Sim K. (2015). The impact of genome wide supported microRNA-137 (MIR137) risk variants on frontal and striatal white matter integrity, neurocognitive functioning, and negative symptoms in schizophrenia. Am. J. Med. Genet. B Neuropsychiatr. Genet..

[72] Ripke S., Sanders A.R., Kendler K.S., Levinson D.F., Sklar P., Holmans P.A., Lin D.Y., Duan J., Ophoff R.A., Andreassen O.A. (2011). Genome-wide association study identifies five new schizophrenia loci. Nat. Genet..

[73] Wei H., Yuan Y., Liu S., Wang C., Yang F., Lu Z., Wang C., Deng H., Zhao J., Shen Y. (2015). Detection of circulating miRNA levels in schizophrenia. Am. J. Psychiatry.

[74] Lai C.Y., Yu S.L., Hsieh M.H., Chen C.H., Chen H.Y., Wen C.C., Huang Y.H., Hsiao P.C., Hsiao C.K., Liu C.M. (2011). MicroRNA expression aberration as potential peripheral blood biomarkers for schizophrenia. PLoS ONE.

[75] Zhao Z.L., Jinde S., Koike S., Tada M., Satomura Y., Yoshikawa A., Nishimura Y., Takizawa R., Kinoshita A., Sakakibara E. (2019). Altered expression of microRNA-223 in the plasma of patients with first-episode schizophrenia and its possible relation to neuronal migration-related genes. Transl. Psychiatry.

[76] Lai C.Y., Lee S.Y., Scarr E., Yu Y.H., Lin Y.T., Liu C.M., Hwang T.J., Hsieh M.H., Liu C.C., Chien Y.L. (2016). Aberrant expression of microRNAs as biomarker for schizophrenia: From acute state to partial remission, and from peripheral blood to cortical tissue. Transl. Psychiatry.

[77] Krutzfeldt J., Rajewsky N., Braich R., Rajeev K.G., Tuschl T., Manoharan M., Stoffel M. (2005). Silencing of microRNAs in vivo with ‘antagomirs’. Nature.

[78] Jimenez-Mateos E.M., Engel T., Merino-Serrais P., McKiernan R.C., Tanaka K., Mouri G., Sano T., O’Tuathaigh C., Waddington J.L., Prenter S. (2012). Silencing microRNA-134 produces neuroprotective and prolonged seizure-suppressive effects. Nat. Med..

[79] Lasda E., Parker R. (2014). Circular RNAs: Diversity of form and function. RNA.

[80] Hansen T.B., Wiklund E.D., Bramsen J.B., Villadsen S.B., Statham A.L., Clark S.J., Kjems J. (2011). miRNA-dependent gene silencing involving Ago2-mediated cleavage of a circular antisense RNA. EMBO J..

[81] Zhong S., Ouyang Q., Zhu D., Huang Q., Zhao J., Fan M., Cai Y., Yang M. (2020). Hsa_circ_0088036 promotes the proliferation and migration of fibroblast-like synoviocytes by sponging miR-140-3p and upregulating SIRT 1 expression in rheumatoid arthritis. Mol. Immunol..

[82] Miao L., Yin R.X., Zhang Q.H., Liao P.J., Wang Y., Nie R.J., Li H. (2019). A novel circRNA-miRNA-mRNA network identifies circ-YOD1 as a biomarker for coronary artery disease. Sci. Rep..

[83] Liu Z., Zhou Y., Liang G., Ling Y., Tan W., Tan L., Andrews R., Zhong W., Zhang X., Song E. (2019). Circular RNA hsa_circ_001783 regulates breast cancer progression via sponging miR-200c-3p. Cell Death Dis..

[84] Wang B., Hua P.Y., Zhao B., Li J.D., Zhang Y. (2020). Circular RNA circDLGAP4 is involved in lung cancer development through modulating microRNA-143/CDK1 axis. Cell Cycle.

[85] Zhou P., Xie W., Huang H.L., Huang R.Q., Tian C., Zhu H.B., Dai Y.H., Li Z.Y. (2020). circRNA_100859 functions as an oncogene in colon cancer by sponging the miR-217-HIF-1alpha pathway. Aging (Albany NY).

[86] Chen H., Pei L., Xie P., Guo G. (2020). Circ-PRKDC Contributes to 5-Fluorouracil Resistance of Colorectal Cancer Cells by Regulating miR-375/FOXM1 Axis and Wnt/beta-Catenin Pathway. OncoTargets Ther..

[87] Che H., Ding H., Jia X. (2020). circ_0080145 Enhances Imatinib Resistance of Chronic Myeloid Leukemia by Regulating miR-326/PPFIA1 Axis. Cancer Biother. Radiopharm..

[88] Li Y., Lin S., An N. (2020). Hsa_circ_0009910: Oncogenic circular RNA targets microRNA-145 in ovarian cancer cells. Cell Cycle.

[89] Hentze M.W., Castello A., Schwarzl T., Preiss T. (2018). A brave new world of RNA-binding proteins. Nat. Rev. Mol. Cell Biol..

[90] Wang Z.L., Li B., Luo Y.X., Lin Q., Liu S.R., Zhang X.Q., Zhou H., Yang J.H., Qu L.H. (2018). Comprehensive Genomic Characterization of RNA-Binding Proteins across Human Cancers. Cell Rep..

[91] Maziuk B., Ballance H.I., Wolozin B. (2017). Dysregulation of RNA Binding Protein Aggregation in Neurodegenerative Disorders. Front. Mol. Neurosci..

[92] Weick J., Rodriguez B., Amoah S., DellOrco M., Hafez A., Hartley B., Brennand K., Haggarty S., Perrone-Bizzozero N., Mellios N. (2019). Dysregulated In Psychiatric Disorders Circular RNAs Interact With Rna Binding Proteins To Regulate Synaptic Plasticity. Eur. Neuropsychopharmacol..

[93] Saito Y., Yuan Y., Zucker-Scharff I., Fak J.J., Jereb S., Tajima Y., Licatalosi D.D., Darnell R.B. (2019). Differential NOVA2-Mediated Splicing in Excitatory and Inhibitory Neurons Regulates Cortical Development and Cerebellar Function. Neuron.

[94] Perycz M., Urbanska A.S., Krawczyk P.S., Parobczak K., Jaworski J. (2011). Zipcode binding protein 1 regulates the development of dendritic arbors in hippocampal neurons. J. Neurosci..

[95] Olesnicky E.C., Antonacci S., Popitsch N., Lybecker M.C., Titus M.B., Valadez R., Derkach P.G., Marean A., Miller K., Mathai S.K. (2018). Shep interacts with posttranscriptional regulators to control dendrite morphogenesis in sensory neurons. Dev. Biol..

[96] Modic M., Ule J., Sibley C.R. (2013). CLIPing the brain: Studies of protein-RNA interactions important for neurodegenerative disorders. Mol. Cell Neurosci..

[97] Zhou Y., Dong F., Mao Y. (2018). Control of CNS functions by RNA-binding proteins in neurological diseases. Curr. Pharmacol. Rep..

[98] Tsuboi D., Kuroda K., Tanaka M., Namba T., Iizuka Y., Taya S., Shinoda T., Hikita T., Muraoka S., Iizuka M. (2015). Disrupted-in-schizophrenia 1 regulates transport of ITPR1 mRNA for synaptic plasticity. Nat. Neurosci..

[99] Millar J.K., Wilson-Annan J.C., Anderson S., Christie S., Taylor M.S., Semple C.A., Devon R.S., St Clair D.M., Muir W.J., Blackwood D.H. (2000). Disruption of two novel genes by a translocation co-segregating with schizophrenia. Hum. Mol. Genet..

[100] Lencz T., Szeszko P.R., DeRosse P., Burdick K.E., Bromet E.J., Bilder R.M., Malhotra A.K. (2010). A schizophrenia risk gene, ZNF804A, influences neuroanatomical and neurocognitive phenotypes. Neuropsychopharmacology.

[101] Zhou Y., Dong F., Lanz T.A., Reinhart V., Li M., Liu L., Zou J., Xi H.S., Mao Y. (2018). Interactome analysis reveals ZNF804A, a schizophrenia risk gene, as a novel component of protein translational machinery critical for embryonic neurodevelopment. Mol. Psychiatry.

[102] Chapman R.M., Tinsley C.L., Hill M.J., Forrest M.P., Tansey K.E., Pardinas A.F., Rees E., Doyle A.M., Wilkinson L.S., Owen M.J. (2019). Convergent Evidence That ZNF804A Is a Regulator of Pre-messenger RNA Processing and Gene Expression. Schizophr. Bull..

[103] Xiao X., Luo X.J., Chang H., Liu Z., Li M. (2017). Evaluation of European Schizophrenia GWAS Loci in Asian Populations via Comprehensive Meta-Analyses. Mol. Neurobiol..

[104] O’Donovan M.C., Craddock N., Norton N., Williams H., Peirce T., Moskvina V., Nikolov I., Hamshere M., Carroll L., Georgieva L. (2008). Identification of loci associated with schizophrenia by genome-wide association and follow-up. Nat. Genet..

[105] Gumina V., Colombrita C., Fallini C., Bossolasco P., Maraschi A.M., Landers J.E., Silani V., Ratti A. (2019). TDP-43 and NOVA-1 RNA-binding proteins as competitive splicing regulators of the schizophrenia-associated TNIK gene. Biochim. Biophys. Acta Gene Regul. Mech..

[106] Huang A., Zheng H., Wu Z., Chen M., Huang Y. (2020). Circular RNA-protein interactions: Functions, mechanisms, and identification. Theranostics.

[107] Wang Z., Lei X. (2020). Matrix factorization with neural network for predicting circRNA-RBP interactions. Bmc Bioinformatics.

[108] Zhang M., Wang T., Xiao G., Xie Y. (2020). Large-Scale Profiling of RBP-circRNA Interactions from Public CLIP-Seq Datasets. Genes.

[109] Dong W., Dai Z.H., Liu F.C., Guo X.G., Ge C.M., Ding J., Liu H., Yang F. (2019). The RNA-binding protein RBM3 promotes cell proliferation in hepatocellular carcinoma by regulating circular RNA SCD-circRNA 2 production. EBioMedicine.

[110] Feng Y., Yang Y., Zhao X., Fan Y., Zhou L., Rong J., Yu Y. (2019). Circular RNA circ0005276 promotes the proliferation and migration of prostate cancer cells by interacting with FUS to transcriptionally activate XIAP. Cell Death Dis..

[111] Du W.W., Yang W., Liu E., Yang Z., Dhaliwal P., Yang B.B. (2016). Foxo3 circular RNA retards cell cycle progression via forming ternary complexes with p21 and CDK2. Nucleic Acids Res..

[112] Dell’Orco M., Oliver R.J., Perrone-Bizzozero N. (2020). HuD Binds to and Regulates Circular RNAs Derived from Neuronal Development- and Synaptic Plasticity-Associated Genes. Front. Genet..

[113] Horvath S., Mirnics K. (2015). Schizophrenia as a Disorder of Molecular Pathways. Biol. Psychiat..

[114] Weickert C.S., Weickert T.W., Pillai A., Buckley P.F. (2013). Biomarkers in schizophrenia: A brief conceptual consideration. Dis. Markers.

[115] Xu C., Jackson S.A. (2019). Machine learning and complex biological data. Genome Biol..

[116] Friedman R.C., Farh K.K., Burge C.B., Bartel D.P. (2009). Most mammalian mRNAs are conserved targets of microRNAs. Genome Res..

[117] Lewis B.P., Burge C.B., Bartel D.P. (2005). Conserved seed pairing, often flanked by adenosines, indicates that thousands of human genes are microRNA targets. Cell.

[118] Xie X., Lu J., Kulbokas E.J., Golub T.R., Mootha V., Lindblad-Toh K., Lander E.S., Kellis M. (2005). Systematic discovery of regulatory motifs in human promoters and 3′ UTRs by comparison of several mammals. Nature.

[119] Hanna J., Hossain G.S., Kocerha J. (2019). The Potential for microRNA Therapeutics and Clinical Research. Front. Genet..

[120] Ruegger S., Grosshans H. (2012). MicroRNA turnover: When, how, and why. Trends Biochem. Sci..

[121] Holdt L.M., Kohlmaier A., Teupser D. (2018). Circular RNAs as Therapeutic Agents and Targets. Front. Physiol..

[122] Zhang Y., Xue W., Li X., Zhang J., Chen S., Zhang J.L., Yang L., Chen L.L. (2016). The Biogenesis of Nascent Circular RNAs. Cell Rep..

[123] Chen W., Schuman E. (2016). Circular RNAs in Brain and Other Tissues: A Functional Enigma. Trends Neurosci..

[124] D’Ambra E., Capauto D., Morlando M. (2019). Exploring the Regulatory Role of Circular RNAs in Neurodegenerative Disorders. Int. J. Mol. Sci..

[125] Lavenniah A., Luu T.D.A., Li Y.Q.P., Lim T.S.B., Jiang J.M., Ackers-Johnson M., Foo R.S.Y. (2020). Engineered Circular RNA Sponges Act as miRNA Inhibitors to Attenuate Pressure Overload-Induced Cardiac Hypertrophy. Mol. Ther..

[126] Zhang M., Xin Y. (2018). Circular RNAs: A new frontier for cancer diagnosis and therapy. J. Hematol. Oncol..

